# Noninvasive models for predicting poor prognosis of chronic HBV infection patients precipitating acute HEV infection

**DOI:** 10.1038/s41598-020-59670-4

**Published:** 2020-02-17

**Authors:** Qiang Li, Chong Chen, Chenlu Huang, Wei Xu, Qiankun Hu, Liang Chen

**Affiliations:** 1 Department of liver disease, Shanghai Public Health Clinical Center, Fudan University, Shanghai, China 201508; 2 Department of Infectious Disease, Shanghai Public Health Clinical Center, Fudan University, Shanghai, China 201508

**Keywords:** Viral hepatitis, Liver diseases, Prognosis

## Abstract

Hepatitis E virus (HEV) infection contributes to a considerable proportion of acute-on-chronic liver failure (ACLF) in patients with chronic hepatitis B virus (HBV) infection. This study aimed to predict the prognosis of chronic HBV infection patients precipitating acute HEV infection. A total of 193 patients were enrolled in this study. The performances of three chronic liver disease prognostic models (CTP score, MELD score, and CLIF-C ADs) were analyzed for predicting the development of ACLF following HEV superimposing chronic HBV infection. Subsequently, the performances of five ACLF prognostic assessment models (CTP score, MELD score, CLIF-C ACLFs, CLIF-C OFs, and COSSH-ACLFs) were analyzed for predicting the outcome of those ACLF patients. Of 193 chronic HBV infection patients precipitating acute HEV infection, 13 patients were diagnosed ACLF on admission, 54 patients developed to ACLF after admission, and 126 patients had non-ACLF during the stay in hospital. For predicting the development of ACLF, CTP score yielded a significantly higher AUROC compared with MELD score and CLIF-C ADs (0.92, 0.88, and 0.86, respectively; all *p* < 0.05). For predicting the poor prognosis of ACLF patients, the COSSH-ACLFs yielded a significantly higher AUROC compared with CLIF-C ACLFs, CLIF-C OFs, MELD score, and CTP score (0.89, 0.83, 0.81, 0.67, and 0.58, respectively; all *p* < 0.05). In conclusion, the stepwise application of CTP score and COSSH-ACLFs can predict the prognosis of chronic HBV infection patients precipitating acute HEV infection.

## Introduction

Infection with hepatitis B virus (HBV) remains a global public health problem, which causes an annual mortality of 780,000 of which 650,000 deaths occur in patients with chronic HBV infection; and 130,000 deaths are a result of acute HBV infection^1^. Hepatitis E virus (HEV) infection is another important public health concern, and hepatitis E is an important contributor to the global viral hepatitis burden^2^. Estimations revealed 20 million populations have been infected with HEV and around 70,000 HEV-related deaths worldwide every year^3^. Because of high incidence, it is easy to observe patients with HEV superimposing HBV infection clinically.

Hepatitis E, when present as a co-infection or super-infection in chronic HBV infection patients, might lead to more severe disease with higher mortality than single hepatitis B. Meanwhile, the underlying chronic HBV infection could predispose the co-infected patients to more severe prognosis than patients with HEV mono-infection. Zhang *et al*. reported that HBV-HEV co-infection represented 20–40% of symptomatic acute HEV infection^4^. Another study by Zhang *et al*. found that nearly 60% of HEV infection patients had underlying liver diseases, including nearly 40% patients with chronic HBV infection^5^.

HEV infection is one of the most common reasons causing acute-on-chronic liver failure (ACLF) in patients with chronic HBV infection in HEV endemic areas^6^. Accumulating evidence suggested that super-infection with HEV triggers ACLF in patients with chronic liver disease^7,8^. Hepatitis E mortality rates of 0.5–4% in the general population, and the rates can exceed 20% in patients with chronic liver disease including chronic HBV infection^9,10^. However, for patients with chronic HBV infection, little is known on which patients would develop ACLF after superimposing HEV infection, and which patients would have poor prognosis following ACLF. In this study, we evaluated the role of six prognostic assessment models in predicating the prognosis of chronic HBV infection patients precipitating acute HEV infection.

## Patients and Methods

### Patients

**Figure 1:**
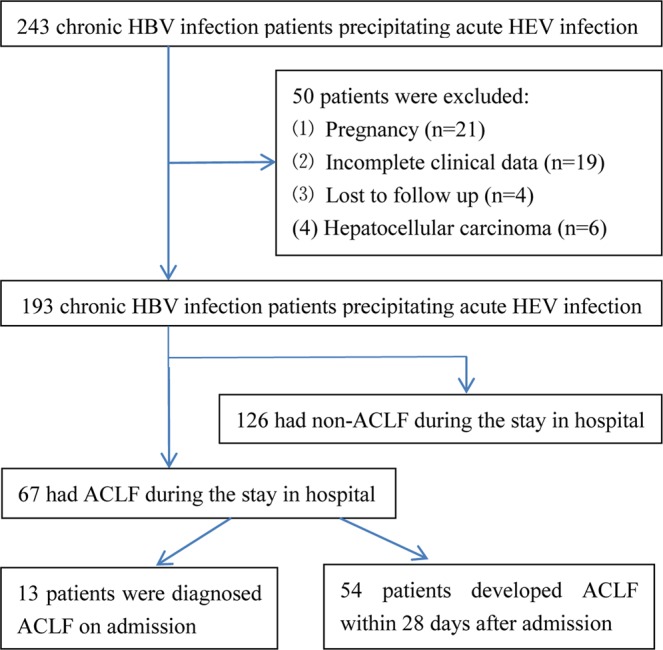
Flow diagram of the study population. HEV, hepatitis E virus; HBV, hepatitis B virus; ACLF, acute-on-chronic liver failure.

A total of 243 chronic HBV infection patients precipitating acute HEV infection from Shanghai Public Health Clinical Center, a tertiary hospital in Shanghai, China, between September 2009 and September 2014, were retrospectively analyzed. The inclusion criteria were as follows: (1) serum HBsAg and/or HBV DNA positivity for more than 6 months; (2) serum anti-HEV immunoglobulin IgM positivity on admission, and seroconverted to serum anti-HEV immunoglobulin IgG positivity during follow-up; (3) had been hospitalized with HEV superimposing chronic HBV infection in Shanghai Public Health Clinical Center. The exclusion criteria were as follows: (1) pregnancy (n = 21), (2) incomplete clinical data (n = 19), (3) lost to follow up (n = 4), (4) hepatocellular carcinoma (n = 6). Finally, 193 chronic HBV infection patients precipitating acute HEV infection were enrolled in this study. The flow chart of enrolled patients was showed in Fig. 1.

The research proposal was approved by the ethics committee of Shanghai Public Health Clinical Center. The clinical diagnosis and treatment process and the research process were all in keeping with the Helsinki declaration. All enrolled patients in this study signed the informed consent on admission.

### Diagnostic criteria

Chronic HBV infection was diagnosed using serum HBsAg and/or HBV DNA positivity for more than six months^11^. Acute HEV infection was diagnosed using two consecutive positive serum anti-HEV immunoglobulin IgM test results, and seroconverted to serum anti-HEV immunoglobulin IgG positivity during follow-up. ACLF was diagnosed according to the consensus recommendations of the Asian Pacific Association for the Study of the Liver (APASL) 2014 for ACLF^12^: jaundice (total bilirubin ≥ 5 mg/dL) and coagulopathy (INR ≥ 1.5) complicated within 4 weeks by ascites and/ or hepatic encephalopathy in a patient with or without previously diagnosed chronic liver disease^12^. The poor prognosis of ACLF was defined as death or liver transplantation within 28 days after admission.

### Clinical laboratory measurements

Serum anti-HEV immunoglobulin IgM and IgG were tested using enzyme-linked immunosorbent assay (ELISA) (MP Biomedicals, Singapore) according to the manufacturers’ instructions. Liver functions were tested using fully-auto-biochemistry-analysis instruments (7600 Series; Hitachi, Japan). Serum HBV markers were detected using ELISA methods (ARCHITECT i2000 SR; Abbott, Germany). HBV viral load quantification was detected using real-time PCR (ABI 7500; Applied Biosystems Inc, United States). Routine blood tests were performed using automated blood cell analyzers (XT-2000i; Sysmex, Japan).

### Choice of prognostic assessment models

Until 2013, Child-Turcotte-Pugh (CTP) score and model for end-stage liver disease (MELD) score were the only two available methods to assess prognosis of ACLF patients. According to the chronic liver failure (CLIF) Consortium of EASL, the CLIF acute decompensation score (CLIF-C ADs) can be used to predict prognosis of hospitalised cirrhotic acute decompensation patients without ACLF^13^. The CLIF-SOFA score was a modified version of SOFA score, proposed by the EASL CLIF Consortium, which can be used to predict the prognosis of ACLF^14^. The CLIF-C OFs is a simplified version of CLIF SOFA score, and has the same performance with CLIF-SOFA score for predicting the prognosis of ACLF^14,15^. The CLIF-Consortium ACLF score (CLIF-C ACLFs) was proposed for predicting the prognosis of ACLF based on two large prospective studies performed by the EASL CLIF Consortium^14,15^. Wu *et al*. developed a prognostic score for HBV-related ACLF, named the Chinese Group on the Study of Severe Hepatitis B (COSSH)-ACLF score (COSSH-ACLFs)^16^. Compared with the EASL-ACLFs, the COSSH-ACLFs identified approximately 20% more ACLF patients^16^.

### Calculation of prognostic assessment models

1. CTP score can be calculated according to five clinical index: total bilirubin (<34 umol/L, 1 point; 34–51 umol/L, 2 points; >51 umol/L, 3 points); albumin (>35 g/L, 1 point; 28–35 g/L, 2 points; <28 g/L, 3 points); ascites (no, 1 point; mild to moderate, 2 points; severe, 3 points); hepatic encephalopathy (no, 1 point; grade 1–2, 2 points; grade 3–4, 3 points); prothrombin time lengthening (1–3 s, 1 point; 4–6 s, 2 points; >6 s, 3 points).
2. MELD score = 96 × Ln Creatinine (mg/dl) + 3.8 × Ln Bilirubin (mg/dl) + 11.2 × Ln (INR) + 6.4 × etiology (1 for viral hepatitis, and 0 for non-viral liver disease)
3. CLIF-C ACLFs can be calculated according to the formula published by Jalan *et al*.^14^: 10 × (0.33 × CLIF-C OFs + 0.04 × age + 0.63 × ln (WBC count) − 2).
4. CLIF-C ADs can be calculated according to the formula published by Jalan *et al*.^13^: 10 × 0.03 × Age (years) + 0.66 × Ln Creatinine (mg/dl) + 1.71 × Ln (INR) + 0.88 × Ln (WBC (10^9^ cells/L)) − 0.05 × Sodium (mmol/L) + 8.
5. COSSH-ACLF = (0.741 × INR + 0.523 × HBV SOFA + 0.026 × age + 0.003 × total bilirubin)^16^.
6. CLIF-SOFA and CLIF-C OFs can be calculated according to Table 1 in the study by Moreau *et al*.^15^ and Table 2 in the study by Jalan *et al*.^14^, respectively.

**Table 1:** Baseline characteristics of enrolled patients and comparison between ACLF group and non-ACLF group.

	Total (n = 193)	ACLF (n = 67)	Non-ACLF (n = 126)	P value
Age (years)	49 ± 14	54 ± 13	47 ± 14	< 0.001
Male n (%)	138 (71.5%)	85 (67.5%)	53 (79.1%)	0.088
ALT (IU/L)	375 (85–893)	310 (81–878)	434 (87–901)	<0.001
AST (IU/L)	196 (80–532)	176 (61–443)	249 (93–659)	0.021
GGT (IU/L)	118 (63–182)	94 (53–138)	135 (69–201)	0.015
TB (mg/dl)	5.64 (1.30–15.55)	16.5 (8.20–25.70)	2.19 (0.97–8.97)	<0.001
ALB (g/L)	36 ± 6	33 ± 6	38 ± 6	<0.001
GLB (g/L)	29 (26–35)	30 (24–37)	29 (26–33)	0.558
Cr (mg/dl)	0.73 (0.62–0.82)	0.76 (0.59–0.87)	0.72 (0.62–0.81)	0.312
WBC (10^9^ cells/L)	5.3 (4.0–6.9)	6.1 (4.5–8.4)	5.1 (3.9–6.3)	0.007
PLT (10^9^ cells/L)	127 ± 63	99 ± 54	141 ± 64	<0.001
INR	1.16 (1.02–1.58)	1.85 (1.53–2.44)	1.08 (0.99–1.19)	<0.001
Cirrhosis, n (%)	71 (36.8%)	42 (62.7%)	29 (23.0%)	<0.001
HE, n (%)	37 (19.2%)	34 (50.7%)	3 (2.4%)	<0.001
Ascites, n (%)	66 (34.2%)	49 (73.1%)	17 (13.5%)	<0.001
CTP score	7 (6–10)	11 (11–13)	6 (5–7)	<0.001
MELD score	12 (5–19)	21 (16–27)	7 (3–13)	<0.001
CLIF-C ADs	40 (35–48)	49 (46–59)	37 (34–41)	<0.001

ACLF, acute-on-chronic liver failure; ALT, alanine aminotransferase; AST, aspartate aminotransferase; GGT, gamma-glutamyl transpeptidase; TB, total bilirubin; ALB, albumin; GLB, globulin; Cr, creatinine; WBC, White blood cell count; PLT, Platelet count; INR, international Normalized Ratio; HE, hepatic encephalopathy; CTP score, Child-Turcotte-Pugh score; MELD score, model for end-stage liver disease score; CLIF-C ADs, Chronic Liver Failure Consortium acute decompensation score.

**Table 2:** Correlation analysis between prognostic models and the outcome of patients.

Prognostic models	Spearman’s r	P value
**Correlation with the development of ACLF**		
CTP score	0.76	<0.001
MELD score	0.64	<0.001
CLIF-C ADs	0.62	<0.001
**Correlation with the poor prognosis of ACLF**		
COSSH-ACLFs	0.72	<0.001
CLIF-C ACLFs	0.66	<0.001
CLIF-C OFs	0.64	<0.001
MELD score	0.52	<0.001
CTP score	0.43	<0.001

ACLF, acute-on-chronic liver failure; Poor prognosis, death or liver transplantation; Spearman’s r, correlation coefficient; CTP score, Child-Turcotte-Pugh score; MELD score, model for end-stage liver disease score; CLIF-C ADs, Chronic Liver Failure Consortium (CLIF) acute decompensation score; COSSH-ACLFs, Chinese Group on the Study of Severe Hepatitis B ACLF score; CLIF-C ACLFs, CLIF ACLF score; CLIF-C OFs, CLIF organ failure score.

### Statistical analysis

All statistical analysis in this study was performed using statistical software SPSS 15.0 (SPSS Inc. USA) and MedCalc 16.1 (MedCalc Software, Belgium). The Kolmogorov-Smirnov test was used to check the normality of data. Data was showed as follows: normal distribution data as mean ± SD, non-normal distribution continuous data as median (IQR), and categorical data as number (percentage). The data between two groups were compared with Chi-squared-test (for categorical data), Mann-Whitney-test (for non-normal continuous data), and t-test (for normal data), respectively. Pearson correlation coefficient was used to perform correlation analysis between two variables. The areas under Receiver Operator Characteristic (ROC) curves (AUROCs) were calculated to evaluate the performances of the prognostic assessment models. The AUROCs were compared using the Delong test^17^. The optimal cut-offs were obtained by maximizing Youden index (sensitivity + specificity − 1). All significance tests were two-tailed, and *p* ≥ 0.05 was considered no significant difference between two groups/methods.

## Results

### Baseline characteristics of enrolled patients

Demographic and biochemical characteristics of the study population were summarized in Table 1. The majority of patients were male (71.5%) and middle-aged (mean 49 years). Of 193 patients, 71 (36.8%) had cirrhosis, 37 (19.2%) had hepatic encephalopathy, and 66 (34.2%) had ascites on admission. The median alanine aminotransferase (ALT), aspartate aminotransferase (AST), gamma-glutamyl transpeptidase (GGT), total bilirubin, globulin, serum creatinine, and INR were 375 IU/L (IQR = 85–893), 196 IU/L (IQR = 80–532), 118 IU/L (IQR = 63–182), 5.64 mg/dl (IQR = 1.30–15.55), 29 g/L (IQR = 26–35), 0.73 mg/dL (IQR = 0.62–0.82), and 1.16 (IQR = 1.02–1.58), respectively. The mean albumin and platelet count levels were 36 g/L and 127 × 10^9^ cells/L, respectively.

### Comparison between ACLF group and non-ACLF group

Of 193 chronic HBV infection patients precipitating acute HEV infection, 13 patients were diagnosed ACLF on admission, 54 patients developed to ACLF within 28 days after admission, and 126 patients had non-ACLF during the stay in hospital. The comparison between ACLF group and non-ACLF group was showed in Table 1. Patients with ACLF had significantly higher age (mean, 54 *vs* 47 years, *p* < 0.001), total bilirubin (median, 16.5 *vs* 2.19 mg/dl, *p* < 0.001), white blood cell count (median, 6.1 *vs* 5.1 × 10^9^ cells/L, *p* = 0.007), INR (median, 1.85 *vs* 1.08, *p* < 0.001), CTP score (median, 11 *vs* 6, *p* < 0.001), MELD score (median, 21 *vs* 7, *p* < 0.001), and CLIF-C ADs (median, 49 *vs* 37, *p* < 0.001); but significantly lower ALT (median, 310 *vs* 434, *p* < 0.001), AST (median, 176 *vs* 249, *p* = 0.021), GGT (median, 94 *vs* 135, *p* = 0.015), and platelet count levels (mean, 99 *vs* 141 × 10^9^ cells/L, *p* < 0.001) compared with patients without ACLF.

### Correlation between prognostic models and the outcome of patients

Based on the fact that only the patients developed ACLF during hospitalization after enrollment could be counted for prediction. First, the correlation analysis was performed between 54 patients developed ACLF within 28 days after admission and 126 patients who had non-ACLF during the stay in hospital. The results showed that the development of ACLF after admission significantly correlated with CTP score (r = 0.76, *p* < 0.001), MELD score (r = 0.64, *p* < 0.001), and CLIF-C ADs (r = 0.64, *p* < 0.001) at the baseline (Table 2). Next, the correlation analysis was performed between 30 ACLF patients with poor prognosis and 37 ACLF patients with favorable outcome. The results showed that the poor prognosis of ACLF significantly correlated with COSSH-ACLFs (r = 0.72, *p* < 0.001), CLIF-C ACLFs (r = 0.66, *p* < 0.001), CLIF-C OFs (r = 0.64, *p* < 0.001), MELD score (r = 0.52, *p* < 0.001), and CTP score (r = 0.43, *p* < 0.001) (Table 2).

### AUROCs comparison of prognostic models

**Figure 2:**
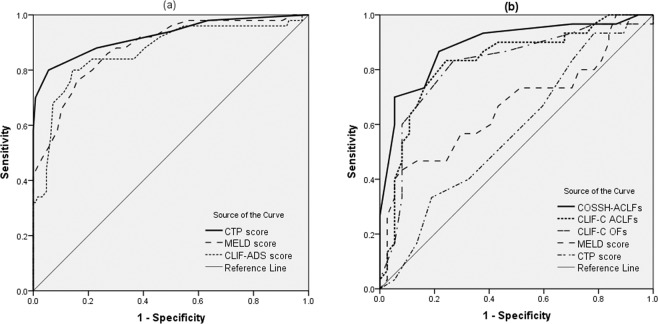
ROC curves for predicting the development of ACLF (**a**) and poor prognosis of ACLF patients (**b**). For predicting the development of ACLF, CTP score had a significantly higher AUROC than MELD score and CLIF-C ADs (0.92, 0.88, and 0.86 for CTP score, MELD score, and CLIF-C ADs, respectively; all *p* < 0.005). For predicting the poor prognosis of ACLF patients, COSSH-ACLFs had a significantly better diagnostic performance than CLIF-C ACLFs, CLIF-C OFs, MELD score, and CTP score (AUROC of 0.89, 0.83, 0.81, 0.67, and 0.58 for COSSH-ACLFs, CLIF-C ACLFs, CLIF-C OFs, MELD score, and CTP score, respectively; all *p < 0.05*).

ROC curve analysis was performed to predict the development of ACLF (a) and poor prognosis (b) of ACLF patients (Fig. 2). Pairwise comparison of AUROCs was presented in Table 3. For predicting the development of ACLF, CTP score had a significantly higher AUROC than MELD score and CLIF-C ADs (0.92, 0.88, and 0.86 for CTP score, MELD score, and CLIF-C ADs, respectively; all *p* < 0.005).

**Table 3:** AUROCs of prognostic models for predicting the development of ACLF.

	The development of ACLF	
	AUROC	(95% CI)
CTP score	0.92	(0.87–0.98)
MELD score	0.88	(0.83–0.94)
CLIF-C ADs	0.86	(0.80–0.93)
CTP score *vs* MELD	*p* = *0.031*	
CTP score *vs* CLIF-C ADs	*P* = *0.045*	

AUROC, area under the receiver operating characteristic curve; ACLF, acute-on-chronic liver failure; CTP score, Child-Turcotte-Pugh score; MELD score, model for end-stage liver disease score; CLIF-C ADs, Chronic Liver Failure Consortium acute decompensation score.

In order to evaluate the ability of prognostic models in predicting the outcome of ACLF patients, we divided the 67 ACLF patients into two groups: favorable outcome group (37 patients) and poor prognosis (liver transplantation/death) group (30 patients). For predicting the poor prognosis of ACLF patients, COSSH-ACLFs had a significantly better diagnostic performance than CLIF-C ACLFs, CLIF-C OFs, MELD score, and CTP score (AUROC of 0.89, 0.83, 0.81, 0.67, and 0.58 for COSSH-ACLFs, CLIF-C ACLFs, CLIF-C OFs, MELD score, and CTP score, respectively; all *p* < *0.05*) (Table 4).

**Table 4:** AUROCs of prognostic models for predicting the poor prognosis of ACLF.

	Poor prognosis of ACLF patients	
	AUROC	(95% CI)
COSSH-ACLFs	0.89	(0.79–0.95)
CLIF-C ACLFs	0.83	(0.72–0.91)
CLIF-C OFs	0.81	(0.70–0.90)
MELD score	0.67	(0.54–0.79)
CTP score	0.58	(0.45–0.70)
COSSH-ACLFs *vs* CLIF-C ACLFs	*p* = *0.016*	
COSSH-ACLFs *vs* CLIF-C OFs	*p* = *0.011*	
COSSH-ACLFs *vs* MELD score	*p* < *0.001*	
COSSH-ACLFs *vs* CTP score	*p* < *0.001*	

Poor prognosis, death or liver transplantation; ACLF, acute-on-chronic liver failure; COSSH-ACLFs, Chinese Group on the Study of Severe Hepatitis B ACLF score; CLIF-C ACLFs, Chronic Liver Failure Consortium ACLF score; CLIF-C OFs, Chronic Liver Failure Consortium organ failure score; MELD score, model for end-stage liver disease score; CTP score, Child-Turcotte-Pugh score.

### Diagnostic thresholds of prognostic models

Diagnostic thresholds of prognostic models were shown in Table 5. For predicting the development of ACLF, the optimal cut-offs were 8 for CTP score (the sensitivity, specificity, PPV, and NPV was 80%, 94%, 85%, and 92%, respectively), 14 for MELD score (the sensitivity, specificity, PPV, and NPV was 76%, 84%, 66%, and 90%, respectively), and 43 for CLIF-ADs (the sensitivity, specificity, PPV, and NPV was 80%, 86%, 69%, and 92%, respectively). For predicting the poor prognosis of ACLF patients, the optimal cut-offs were 10 for COSSH-ACLFs (the sensitivity, specificity, PPV, and NPV was 87%, 78%, 77%, and 88%, respectively), 41 for CLIF-C ACLFs (the sensitivity, specificity, PPV, and NPV was 83%, 76%, 74%, and 85%, respectively), and 11 for CLIF-C OFs (the sensitivity, specificity, PPV, and NPV was 83%, 73%, 71%, and 84%, respectively).

**Table 5:** Diagnostic thresholds of prognostic models.

	Cut-offs	Se (%)	Sp (%)	PPV (%)	NPV (%)	+LR	−LR
**For development of ACLF**							
CTP score	8	80	94	85	92	14.4	0.21
MELD score	14	76	84	66	90	4.79	0.29
CLIF-ADs	43	80	86	69	92	5.56	0.23
**For poor prognosis of ACLF**							
COSSH-ACLFs	10	87	78	77	88	4.01	0.17
CLIF-C ACLFs	41	83	76	74	85	3.43	0.22
CLIF-C OFs	11	83	73	71	84	3.08	0.23

ACLF, acute-on-chronic liver failure; the optimal cut-off points were determined by maximizing Youden index; Se, sensitivity; Sp, specificity; PPV, positive predictive value; NPV, negative predictive value; +LR, positive likelihood ratio; −LR, negative likelihood ratio.

## Discussion

In China, the rate of HBsAg positivity is 7.18% in 2006, and 6.0% in 2016^18^. Meanwhile, recent study showed that the rate of HEV infection is 20%-40% in China^19^. Because of the high prevalence, HEV superimposing HBV infection is not rare in China. The majority of HEV infection patients may not have any clinical symptoms. However, a small percentage of HEV infection may lead to a life-threatening disease, particularly in patients with chronic liver disease^20^. The HEV super-infection has been reported as the second leading cause of ACLF in chronic HBV infection patients^4,21,22^. Kumar *et al*. reported that patients with HEV superimposing HBV infection had more likely to progress to ACLF^8^.

In this study, we showed the proportion of patients developed ACLF in patients following HEV superimposing chronic HBV infection, and then evaluated the prognosis of chronic HBV infection patients precipitating acute HEV infection. Our results showed that 67/193 (34.7%) chronic HBV infection patients developed ACLF following acute HEV infection, and 30/67 (44.8%) of those ACLF patients had poor prognosis. Our results suggested that HEV superimposing HBV infection is a precipitating event to initiate ACLF. Acute HEV infection may be related to the development of ACLF and poor prognosis of patients in patients who have underlying chronic HBV infection.

For chronic HBV infection patients precipitating acute HEV infection, the first question need to be resolved is which patients would develop ACLF. In this study, three prognostic assessment models (CTP score, MELD score, and CLIF-C ADs) were compared for predicting the development of ACLF in chronic HBV infection patients precipitating acute HEV infection. We found that CTP score, MELD score, and CLIF-C ADs were higher in patients developed ACLF, compared with patients had non-ACLF. For predicting the development ACLF, the CTP score yielded a significantly higher performance compared with MELD score and CLIF-C ADs. For those patients, the second question need to be resolved is which patients would have poor prognosis after the development of ACLF. In our study, five prognostic assessment models were analyzed for predicting the poor prognosis of those ACLF patients. We found that COSSH-ACLFs yielded a significantly more diagnostic performance compared with CLIF-C ACLFs, CLIF-C OFs, MELD score, and CTP score for predicting the poor outcome of ACLF patients following HEV superimposing chronic HBV infection.

Therefore, we proposed stepwise application of CTP score and COSSH-ACLFs to predict the outcome of chronic HBV infection patients precipitating acute HEV infection. The CTP score is the best model for predicting the development of ACLF in chronic HBV infection patients precipitating acute HEV infection. The COSSH-ACLFs is the best model for predicting the poor outcome of ACLF patients. The CTP-COSSH-ACLFs algorithm, which sequentially combines CTP-score and COSSH-ACLFs, can discriminate patients at low risk of developing ACLF with excellent prognosis from those at high risk of developing ACLF with impaired prognosis and need specialized care.

Cirrhosis is considered prerequisite for ALCF in Europe and America; however, in Asia, it is considered that ACLF can develop in patients without cirrhosis, including chronic HBV infection patients^12^. In this study, of 67 ACLF patients, 42 (62.7%) had cirrhosis background, 25 (37.3%) had no cirrhosis background. The EASL and AASLD ACLF definitions were proposed and validated only in patients with cirrhosis from Europe and North America, where alcoholic liver disease is the major aetiology^23^. However, in the Asia-Pacific regions including China, the major aetiology of ACLF is HBV infection. Therefore, we defined ACLF according to the ACLF consensus recommendations of the APASL 2014^12^.

Of course, several limitations in this study should be noticed. First, this study is a retrospective single-center study. The results in this study need to be further validated in a large sample, multi-center, and perspective study. Second, this study was performed in a tertiary hospital with a high percentage of seriously ill patients than in the general population. The spectrum bias may appear when extrapolating the results of this study to general population^24,25^. Third, HEV genotyping were not performed in this study. Although we did not detect the HEV genotypes of patients in this cohort, we had reason to believe that the genotypes of almost patients in this study were genotype 4 HEV. Over the past twenty years, HEV genotype 1 has been replaced by HEV genotype 4 as the most common genotype (over 80% of HEV infection patients) in China^26^.

In conclusion, the stepwise application of CTP score and COSSH-ACLFs can predict the outcome of chronic HBV infection patients precipitating acute HEV infection. The CTP-COSSH-ACLFs algorithm provides a method that contributed to regulate the large flow of patients between primary health care institutions and tertiary hospitals: patients had no ACLF tendency can be treatment in primary health care institutions, whereas those had ACLF tendency will need to be redirected to a tertiary hospital for specialized management.
